# Supplementary material for: Heparin-based hydrogel scaffolding alters the transcriptomic profile and increases the chemoresistance of MDA-MB-231 triple-negative breast cancer cells
Source: Biomater Sci. 2020 Feb 13;8(10):2786–96. doi: 10.1039/c9bm01481k (PMC7497406; doi:10.1039/c9bm01481k)
Supplement: Supplementary file 2 [file BM-008-C9BM01481K-s002.zip › Supplementary File 4/HP-BhydrogelvControl/Pathways/my_analysis.Gsea.1545201079907/HALLMARK_MYC_TARGETS_V2.html]

Details for gene set HALLMARK\_MYC\_TARGETS\_V2[GSEA]

|  || Dataset | expr.class.cls#HYD\_versus\_CONTROL.class.cls#HYD\_versus\_CONTROL\_repos |
| Phenotype | class.cls#HYD\_versus\_CONTROL\_repos |
| Upregulated in class | HYD |
| GeneSet | HALLMARK\_MYC\_TARGETS\_V2 |
| Enrichment Score (ES) | 0.30626315 |
| Normalized Enrichment Score (NES) | 1.0484173 |
| Nominal p-value | 0.37765205 |
| FDR q-value | 0.7945501 |
| FWER p-Value | 1.0 |
Table: GSEA Results Summary

  

Fig 1: Enrichment plot: HALLMARK\_MYC\_TARGETS\_V2      
 Profile of the Running ES Score & Positions of GeneSet Members on the Rank Ordered List

  

| PROBE | DESCRIPTION (from dataset) | GENE SYMBOL | GENE\_TITLE | RANK IN GENE LIST | RANK METRIC SCORE | RUNNING ES | CORE ENRICHMENT || 1 | PPRC1 | na |  |  | 384 | 2.505 | 0.0475 | Yes |
| 2 | SLC19A1 | na |  |  | 752 | 2.073 | 0.0842 | Yes |
| 3 | RRP9 | na |  |  | 1722 | 1.481 | 0.0735 | Yes |
| 4 | NOP2 | na |  |  | 1860 | 1.420 | 0.1046 | Yes |
| 5 | IPO4 | na |  |  | 2106 | 1.312 | 0.1272 | Yes |
| 6 | RRP12 | na |  |  | 2114 | 1.308 | 0.1621 | Yes |
| 7 | GRWD1 | na |  |  | 2299 | 1.237 | 0.1858 | Yes |
| 8 | MAP3K6 | na |  |  | 2450 | 1.190 | 0.2101 | Yes |
| 9 | TCOF1 | na |  |  | 2765 | 1.096 | 0.2232 | Yes |
| 10 | MYBBP1A | na |  |  | 2787 | 1.089 | 0.2515 | Yes |
| 11 | NOC4L | na |  |  | 3575 | 0.900 | 0.2346 | Yes |
| 12 | MYC | na |  |  | 3771 | 0.854 | 0.2475 | Yes |
| 13 | PUS1 | na |  |  | 3780 | 0.853 | 0.2701 | Yes |
| 14 | NOP16 | na |  |  | 4286 | 0.755 | 0.2641 | Yes |
| 15 | PPAN | na |  |  | 4616 | 0.691 | 0.2655 | Yes |
| 16 | BYSL | na |  |  | 4766 | 0.665 | 0.2757 | Yes |
| 17 | SLC29A2 | na |  |  | 4773 | 0.664 | 0.2932 | Yes |
| 18 | NOP56 | na |  |  | 4859 | 0.647 | 0.3063 | Yes |
| 19 | WDR74 | na |  |  | 5691 | 0.526 | 0.2770 | No |
| 20 | TBRG4 | na |  |  | 6008 | 0.483 | 0.2736 | No |
| 21 | SRM | na |  |  | 6386 | 0.433 | 0.2656 | No |
| 22 | RABEPK | na |  |  | 7008 | 0.359 | 0.2428 | No |
| 23 | IMP4 | na |  |  | 7610 | 0.291 | 0.2193 | No |
| 24 | FARSA | na |  |  | 7887 | 0.263 | 0.2119 | No |
| 25 | MRTO4 | na |  |  | 7949 | 0.255 | 0.2156 | No |
| 26 | PES1 | na |  |  | 7971 | 0.251 | 0.2213 | No |
| 27 | UNG | na |  |  | 8156 | 0.239 | 0.2181 | No |
| 28 | MCM5 | na |  |  | 8383 | 0.209 | 0.2119 | No |
| 29 | PRMT3 | na |  |  | 8633 | 0.188 | 0.2040 | No |
| 30 | MCM4 | na |  |  | 8791 | 0.168 | 0.2003 | No |
| 31 | NOLC1 | na |  |  | 9142 | 0.135 | 0.1857 | No |
| 32 | AIMP2 | na |  |  | 9143 | 0.135 | 0.1893 | No |
| 33 | RCL1 | na |  |  | 9619 | 0.086 | 0.1668 | No |
| 34 | DCTPP1 | na |  |  | 9940 | 0.047 | 0.1514 | No |
| 35 | TFB2M | na |  |  | 10433 | 0.003 | 0.1257 | No |
| 36 | UTP20 | na |  |  | 10445 | 0.002 | 0.1252 | No |
| 37 | EXOSC5 | na |  |  | 10572 | -0.013 | 0.1190 | No |
| 38 | NDUFAF4 | na |  |  | 10580 | -0.014 | 0.1190 | No |
| 39 | PLK1 | na |  |  | 10895 | -0.053 | 0.1041 | No |
| 40 | PA2G4 | na |  |  | 11816 | -0.152 | 0.0601 | No |
| 41 | CBX3 | na |  |  | 12874 | -0.267 | 0.0121 | No |
| 42 | LAS1L | na |  |  | 12906 | -0.272 | 0.0178 | No |
| 43 | DDX18 | na |  |  | 13554 | -0.361 | -0.0063 | No |
| 44 | WDR43 | na |  |  | 13628 | -0.375 | -0.0000 | No |
| 45 | PLK4 | na |  |  | 13826 | -0.410 | 0.0008 | No |
| 46 | NIP7 | na |  |  | 13870 | -0.414 | 0.0097 | No |
| 47 | HSPE1 | na |  |  | 14343 | -0.470 | -0.0023 | No |
| 48 | HSPD1 | na |  |  | 14793 | -0.536 | -0.0113 | No |
| 49 | GNL3 | na |  |  | 14904 | -0.551 | -0.0022 | No |
| 50 | HK2 | na |  |  | 15767 | -0.695 | -0.0285 | No |
| 51 | PHB | na |  |  | 16365 | -0.825 | -0.0375 | No |
| 52 | CDK4 | na |  |  | 16963 | -0.974 | -0.0424 | No |
| 53 | SUPV3L1 | na |  |  | 17567 | -1.183 | -0.0420 | No |
| 54 | MPHOSPH10 | na |  |  | 17618 | -1.206 | -0.0121 | No |
| 55 | TMEM97 | na |  |  | 17777 | -1.277 | 0.0141 | No |
| 56 | SORD | na |  |  | 18930 | -2.228 | 0.0140 | No |
Table: GSEA details [plain text format]

  

Fig 2: HALLMARK\_MYC\_TARGETS\_V2      
 Blue-Pink O' Gram in the Space of the Analyzed GeneSet

  

Fig 3: HALLMARK\_MYC\_TARGETS\_V2: Random ES distribution      
 Gene set null distribution of ES for **HALLMARK\_MYC\_TARGETS\_V2**
